# Asymptomatic Malaria Infection Is Maintained by a Balanced Pro- and Anti-inflammatory Response

**DOI:** 10.3389/fmicb.2020.559255

**Published:** 2020-11-17

**Authors:** Augustina Frimpong, Jones Amponsah, Abigail Sena Adjokatseh, Dorothy Agyemang, Lutterodt Bentum-Ennin, Ebenezer Addo Ofori, Eric Kyei-Baafour, Kwadwo Akyea-Mensah, Bright Adu, Gloria Ivy Mensah, Linda Eva Amoah, Kwadwo Asamoah Kusi

**Affiliations:** ^1^West African Centre for Cell Biology of Infectious Pathogens (WACCBIP), University of Ghana, Accra, Ghana; ^2^Department of Immunology, Noguchi Memorial Institute for Medical Research, College of Health Sciences, University of Ghana, Accra, Ghana; ^3^African Institute for Mathematical Sciences, Accra, Ghana; ^4^Department of Biochemistry, Cell and Molecular Biology, College of Basic and Applied Sciences, University of Ghana, Accra, Ghana; ^5^Department of Bacteriology, Noguchi Memorial Institute for Medical Research, College of Health Sciences, University of Ghana, Accra, Ghana

**Keywords:** microscopic, *Plasmodium*, anti-inflammatory cytokines, pro-inflammatory cytokines, asymptomatic malaria, submicroscopic

## Abstract

**Background:**

Pro- and anti-inflammatory cytokines are important mediators of immunity and are associated with malaria disease outcomes. However, their role in the establishment of asymptomatic infections, which may precede the development of clinical symptoms, is not as well-understood.

**Methods:**

We determined the association of pro and anti-inflammatory cytokines and other immune effector molecules with the development of asymptomatic malaria. We measured and compared the plasma levels of pro-inflammatory mediators including tumor necrosis factor-alpha (TNF-α), interferon-gamma (IFN-γ), interleukin (IL)-6, IL-12p70, IL-17A, and granzyme B, the anti-inflammatory cytokine IL-4 and the regulatory cytokine IL-10 from children with asymptomatic malaria infections (either microscopic or submicroscopic) and uninfected controls using Luminex.

**Results:**

We show that individuals with microscopic asymptomatic malaria had significantly increased levels of TNF-α and IL-6 compared to uninfected controls. Children with either microscopic or submicroscopic asymptomatic malaria exhibited higher levels of IFN-γ, IL-17A, and IL-4 compared to uninfected controls. The levels of most of the pro and anti-inflammatory cytokines were comparable between children with microscopic and submicroscopic infections. The ratio of IFN-γ/IL-10, TNF-α/IL-10, IL-6/IL-10 as well as IFN-γ/IL-4 and IL-6/IL-4 did not differ significantly between the groups. Additionally, using a principal component analysis, the cytokines measured could not distinguish amongst the three study populations. This may imply that neither microscopic nor submicroscopic asymptomatic infections were polarized toward a pro-inflammatory or anti-inflammatory response.

**Conclusion:**

The data show that asymptomatic malaria infections result in increased plasma levels of both pro and anti-inflammatory cytokines relative to uninfected persons. The balance between pro- and anti-inflammatory cytokines are, however, largely maintained and this may in part, explain the lack of clinical symptoms. This is consistent with the generally accepted observation that clinical symptoms develop as a result of immunopathology involving dysregulation of inflammatory mediator balance in favor of pro-inflammatory mediators.

## Introduction

Malaria is a protozoan infectious disease that puts more than 3 billion of the world’s population at risk (WHO, 2018). Infection with *Plasmodium* may cause different manifestations of the disease based on several factors, including the quality of host acquired immunity. Manifestations of the disease range from microscopic or submicroscopic asymptomatic infections to symptomatic uncomplicated complicated malaria. A significant number of parasite-infected persons in malaria-endemic areas are asymptomatic (Bousema et al., 2014; Snow et al., 2017). These asymptomatic infections are loosely defined as individuals who present with parasites over a period of time but have no clinical symptoms of the disease and have not recently been treated with anti-malarial drugs (Lindblade et al., 2013). These infections manifest as a result of repeated exposure to the parasite over a period resulting in the acquisition of anti-disease immunity. In addition, tolerance to the infection may be multi-factorial based on parasite and host factors (Laishram et al., 2012; Galatas et al., 2016). Nevertheless, investigating the immunological mechanisms that explain asymptomatic infections remains important in order to understand disease etiology (Langhorne et al., 2008; Wammes et al., 2013). Aside the presence of parasites in the host, a significant portion of the clinical symptoms of the disease are believed to be caused by parasite-induced host immune responses, typically those that promote inflammation (Othoro et al., 1999; Prakash et al., 2006).

Naturally acquired antibody responses to key parasite antigens have been noted to play significant roles in the acquisition of anti-malarial immunity despite being shown to be short-lived (White et al., 2014; Partey et al., 2018). In addition, cellular responses to malaria, which may involve various lymphocyte subsets and the cytokines they secrete, have been found to either mediate protection or impede the acquisition of immunity by down-regulating protective immune responses (Frimpong et al., 2018, 2019; Nlinwe et al., 2018; Kurup et al., 2019).

These cytokines may also be secreted by other immune cell subsets such as monocytes or macrophages. Pro-inflammatory cytokines such as IFN-γ and TNF-α produced by Th1 cells are signaling molecules that also help in the recruitment of other cell subsets such as monocytes to phagocytose infected erythrocytes (Akdis et al., 2011). In addition, IL-12p70 produced mainly by macrophages and dendritic cells aid in the activation of T cells and polarizing the immune response to pro-inflammatory responses (Kumar et al., 2019). Also, other pro-inflammatory cytokines like IL-17A produced by Th17 cells and other cell types have been observed to be upregulated during *P. vivax* infection and aid in the recruitment of neutrophils to sites of inflammation (Bueno et al., 2012). In malaria, an early increase in the levels of these pro-inflammatory cytokines has been associated with parasite clearance and plays an important role in resistance to infections (Angulo and Fresno, 2002; Ibitokou et al., 2014). Nevertheless, subsequent uncontrolled levels have been associated with the development of immunopathology which is often found in severe forms of the disease (Mshana et al., 1991; Gimenez et al., 2003; Mackintosh et al., 2004). Therefore, to circumvent the impact of high levels of pro-inflammatory cytokines, anti-inflammatory cytokines such as IL-4, IL5, and IL-13 are secreted to regulate inflammation (Torre et al., 2002). Also, the immunoregulatory cytokines IL-10 and tumor growth factor-beta (TGF-β) have been shown to regulate the levels of both pro- and anti-inflammatory mediators in an effort to restore homeostatic balance (Kumar et al., 2020). On these basis, several studies have proposed that the ratio of pro-inflammatory to anti-inflammatory cytokines as significant predictors of disease outcome (Kurtzhals et al., 1998; Othoro et al., 1999; Kidd, 2003; Sinha et al., 2010; Farrington et al., 2017). While these are well-described for individuals who are symptomatic (Mshana et al., 1991; Kurtzhals et al., 1998; Gimenez et al., 2003; Farrington et al., 2017), there have been no distinctive studies on characterizing the ratio of pro-inflammatory:anti-inflammatory cytokines to determine if asymptomatic infections, whether microscopic or submicroscopic, are polarized by pro-inflammatory or anti-inflammatory responses.

In addition, previous studies have associated asymptomatic malaria with relatively decreased levels of pro-inflammatory responses, relative to the regulatory cytokine IL-10 (Wilson et al., 2010; Ibitokou et al., 2014; de Jong et al., 2017). In pregnant women, it was observed that high levels of IL-10 was a good predictor of infection and maternal anemia (Ibitokou et al., 2014). IL-10 is an immunoregulatory cytokine produced by Th1, Th2, B cells, and some innate cells (Wilson et al., 2010; Ibitokou et al., 2014; de Jong et al., 2017). This regulatory cytokine plays important role in regulating the detrimental effect of most pro-inflammatory cytokines such as TNF-α and IL-12 in a dose-dependent manner and being a significant predictor of parasitemia levels (Kumar et al., 2019). Despite these findings which have led to the postulation that asymptomatic malaria is largely associated with anti-inflammatory responses (Ochola-Oyier et al., 2019), others have reported a suppressed regulatory immune response in asymptomatic malaria (Wammes et al., 2013; de Jong et al., 2017). These together imply that understanding the immune mechanisms associated with asymptomatic malaria needs to be investigated.

In this study, we tested the hypothesis that asymptomatic infections are characterized by increased levels of regulatory or anti-inflammatory cytokines compared to the levels of pro-inflammatory mediators. We determined the levels of pro- and anti-inflammatory mediators as well as that of the regulatory cytokine IL-10 in children with microscopic or submicroscopic asymptomatic malaria and compared to levels in uninfected controls. Additionally, we estimated pro-inflammatory: anti-inflammatory cytokine ratios to determine whether asymptomatic infections are skewed toward any one of these inflammatory mediators.

## Materials and Methods

### Ethics Statement

Ethical approval for the study was obtained from the Institutional Review Board at the Noguchi Memorial Institute for Medical Research, University of Ghana (No. 089/14-15). Written informed consent and assent were properly obtained from participants before sample collection.

### Study Site

Participants for the study were recruited from Obom, a semi-rural community in the Ga South Municipality of the Greater Accra Region of Ghana. Malaria transmission in the community is perennial with peak transmission occurring from May to September. The parasite prevalence in the area as estimated by microscopy is about 35% (Amoah et al., 2016).

### Study Design and Sample Collection

This was a cross-sectional study where archived samples obtained from community children under 15 years of age were used. About 5 ml of peripheral venous blood was collected from each volunteer into EDTA tubes. Parasite density was determined using Giemsa-stained thick blood smears by counting the number of parasites per 200 white blood cell counts. Samples were processed and plasma aliquots were stored in Eppendorf tubes at −80°C until needed for the experiment. Participants who were positive for *Plasmodium* infection via microscopy but showed no clinical symptoms were classified as having microscopic asymptomatic infections, whereas, children who were negative via microscopy but positive via PCR were classified as having a submicroscopic asymptomatic infection. Additionally, children who were negative for microscopy and PCR were classified as uninfected and used as controls.

### Identification of Submicroscopic Infections

Genomic DNA was extracted using the Zymo DNA Kit (Zymo Research, Irvine, United States) according to the manufacturer’s protocol for whole blood samples. Briefly, 100 μl of whole blood was lysed with 400 μl of lysis buffer before running over the spin column. The DNA was eluted using 100 μl of elution buffer and stored at 4°C for immediate use.

The *P. falciparum* 18s rRNA was amplified from 20 to 40 ng of the extracted DNA in a 15 μl reaction volume. The PCR reaction was made up of 2.5 mM MgCl_2_, 200 nM deoxynucleoside triphosphate mix (dNTPs), 1 U of Onetaq^TM^, DNA polymerase (NEB, United Kingdom) and 250 nM each of forward and reverse primers (rPLUS and rPLU; Ayanful-Torgby et al., 2018) and rFAL1(F) and rFAL2(R) for the nested reaction (Adjah et al., 2018). Genomic DNA from 3D7 strain from *P. falciparum* (MRA 102G) and double distilled water was used as positive and negative controls, respectively, for the amplification.

The PCR amplification products were resolved on 2.0% agarose gels stained with 0.5 μg/ml ethidium bromide. The gels were observed under ultraviolet light after electrophoresis. The gel image was captured using Vilber Lourmat Gel Dock System (Vilber Wielandstrasse, Germany).

### Multiplex Immunoassay

Plasma samples were defrosted on ice. Plasma was diluted twofold for the assay. The levels of cytokines were determined using a magnetic bead-based multiplex assay, which enables the quantification of multiple cytokines/analytes in the same plasma sample using a 96 well plate format. A human 8-plex assay kit was used to quantify levels of pro-inflammatory mediators (granzyme B, IFN-γ,-6, IL-12p70, IL-17A, and TNF-α), the anti-inflammatory cytokine IL-4 and the regulatory cytokine IL-10 (R&D Systems, United States). Sample dilutions, reagents, and standards were all prepared according to the manufacturer’s protocol. All blanks and standards were prepared in duplicate on each plate to determine uniformity in the assay. The plates were read using the LUMINEX^®^ 200^TM^ system, running on the Xponent 3.1 software. Analyte levels were reported as the median fluorescent intensity values.

### Statistical Analysis

All data analyses were performed using the Prism version 6.01 (GraphPad Software, Inc.) and the R statistical software version 3.5.2 (R Foundation for Statistical Computing). Categorical data variables were analyzed using the Chi-square test. Continuous variables were analyzed using Kruskal–Wallis or One-way ANOVA, for data involving three groups, whereas, Mann–Whitney *U*-test was used for data comparison between two groups. Both Kruskal–Wallis and Mann–Whitney *U*-tests were used to analyze data that were not normally distributed. Comparison between three groups was followed by a Dunn’s *post-hoc* test or a Bonferroni test to correct for multiple comparisons. The association between cytokines was determined using Spearman’s rank correlation test. Multivariate analyses were performed using a generalized additive model using the mgcv package in R (Wood and Wood, 2015) with a likelihood ratio test to determine cytokines that were predictive of age and parasitemia. A principal component analysis was used to determine if the cytokine profiles could be used to distinguish the study population; uninfected children, children who had microscopic or submicroscopic infection. Statistical significance was set at *P* < 0.05.

## Results

### Demographic and Baseline Characteristics of Participants

Samples from a total of 78 participants, consisting of 18 uninfected controls and 60 children with asymptomatic malaria infections (22 with submicroscopic and 38 microscopic parasite densities) were used in this study. After diagnosis via microscopy and PCR, the proportion of males to females was not different among participants in the various groups (*p* = 0.84). The age distribution differed significantly between the study cohorts (*p* = 0.021); children with submicroscopic asymptomatic malaria were significantly older than uninfected children (*p* = 0.017) but not children with microscopic asymptomatic malaria. Also, hemoglobin levels were comparable among the study participants (*p* = 0.98). Likewise, no statistically significant difference was observed in the temperatures measured among the participants in the various groups (Table 1).

**TABLE 1:** Demographic and clinical characteristics of the study participants.

Characteristics	Controls	Submicroscopic	Microscopic	*P*-value
Sample size (n)	18	22	38	
Sex (n)				
Male	8	11	16	0.84^*a*^
Female	10	11	22	
Age (IQR) years	9 (6.75–11)	11.5 (9–13.25)	10.5 (9–12)	**0.021**^*b*^
Parasitemia (IQR), /μl	NA	NA	935.5 (269.3–1990)	NA
Hemoglobin, level (IQR), g/dl	10.85 (10.68–12.0)	11.05 (10.65–12.03)	11.05 (10.5–12.7)	0.98^*b*^
Temperature (IQR), °C	36.8 (36.55–36.85)	36.80 (36.6–36.9)	36.9 (36.6–36.9)	0.49^*b*^

*IQR, interquartile range; NA, not applicable. ^a^Chi square test. ^b^Kruskal–Wallis test.*

### Elevated Levels of TNF-α and IL-6 in Children With Microscopic Asymptomatic Malaria

**FIGURE 1:**
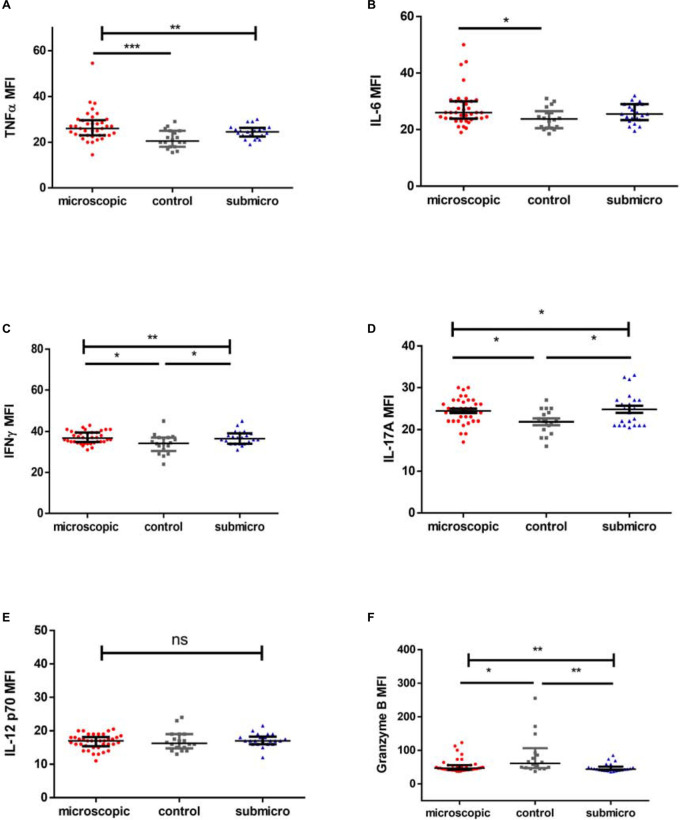
Profile of pro-inflammatory mediators during microscopic and submicroscopic malaria. Scatter plot graphs are plotted showing the median fluorescence intensities (MFI) of **(A–F)** TNF-α, IL-6, IFN-γ, IL-17A, IL-12p70, and Granzyme B in plasma samples collected from uninfected controls (*n* = 18), patients with microscopic asymptomatic malaria (*n* = 38) and submicroscopic malaria (*n* = 22). Plots show median and interquartile ranges. Significant differences are denoted by *p < 0.05, **p < 0.01, ***p < 0.001, ns = not significant.

High levels of pro-inflammatory cytokines have been associated with the development of clinical disease whereas increased levels of IL-6 have been observed in submicroscopic malaria in pregnancy. In this study we observed that the levels of pro-inflammatory cytokines TNF-α and IL-6 were increased significantly only in children with microscopic asymptomatic malaria compared to uninfected controls (*p* = 0.0006; *p* = 0.027, respectively), whereas, levels in children with microscopic parasitemia were comparable to levels in children with submicroscopic parasitemia. Also, levels of both TNF-α and IL-6 in children with submicroscopic infections were comparable to levels in uninfected controls (*p* > 0.05; Figures 1A,B).

### Levels of IFN-γ and IL-17A Are Increased in Children With Microscopic and Submicroscopic Asymptomatic Malaria

For infected children with either microscopic or submicroscopic asymptomatic malaria, levels of the pro-inflammatory cytokine IFN-γ were significantly higher compared to levels in uninfected controls (Figure 1C). However, levels in children with microscopic asymptomatic parasitemia did not differ significantly from levels in children with submicroscopic asymptomatic parasitemia (*p* > 0.05). Similarly, levels of the pro-inflammatory mediator IL-17A (Figure 1D) were increased significantly in children with microscopic and submicroscopic infections compared to controls (*p* = 0.027 and *p* = 0.027, respectively).

Meanwhile, levels of IL-12p70 did not differ between any of the groups (*p* = 0.53; Figure 1E), whereas, levels of granzyme B were found to be significantly lower in children with microscopic (*p* = 0.027) and submicroscopic (*p* = 0.003; Figure 1F) parasitemia compared to uninfected controls.

### Increased Levels of IL-4 in Children With Microscopic and Submicroscopic Asymptomatic Malaria

**FIGURE 2:**
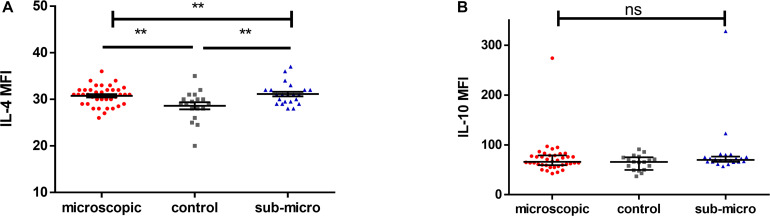
Evaluating the levels of anti-inflammatory cytokines. Scatter plots showing the median fluorescence intensities (MFI) of **(A,B)** IL-4 and IL-10 in plasma samples collected from uninfected controls (*n* = 18) and patients with microscopic asymptomatic malaria (*n* = 38) and submicroscopic malaria (*n* = 22). Plots show median and interquartile ranges. Significant differences are denoted by *p* < 0.01**, ns = not significant.

The levels of anti-inflammatory cytokines, IL-4 and IL-10 in children with microscopic asymptomatic malaria, submicroscopic asymptomatic malaria and uninfected controls were compared (Figure 2). It was observed that levels of IL-4 were increased significantly in children with either microscopic or submicroscopic asymptomatic malaria compared to uninfected controls (*p* = 0.006; *p* = 0.006) However, levels of IL-4 were comparable between children with microscopic and submicroscopic asymptomatic infections (*p* = 0.57). For levels of IL-10, there was no significant difference between the various groups (*p* = 0.23).

### IL-6 and IL-10 Are Major Predictors of Parasitemia in Children With Microscopic Asymptomatic Malaria

**FIGURE 3:**
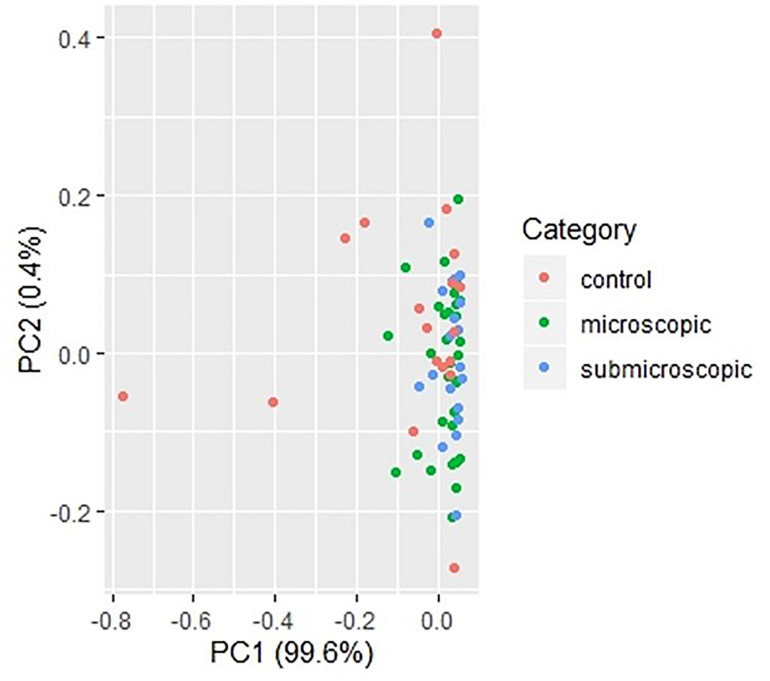
Principal component analysis of cytokine responses in children with microscopic, submicroscopic infections, and uninfected controls. A principal component analysis of cytokine profiles in uninfected (*n* = 18), microscopic asymptomatic infection (*n* = 38) and submicroscopic asymptomatic infected (*n* = 22) children. Uninfected controls are indicated with the red dots, microscopic with green dots and submicroscopic asymptomatic malaria with blue dots.

To identify cytokines that may correlate with each other during infection, we compared the interrelationship between the cytokines in both microscopic (Table 2) and submicroscopic asymptomatic malaria (Supplementary Table S1). For children with microscopic asymptomatic malaria, IL-6 was found to positively correlate with TNF-α (*r* = 0.59, *p* < 0.0001), IL-4 (*r* = 0.39, *p* = 0.017), and IL-10 (*r* = 0.50, *p* = 0.002). Similarly, TNF-α also positively correlated with anti-inflammatory cytokines IL-4 (*r* = 0.41, *p* = 0.011) and IL-10 (*r* = 0.39, *p* = 0.016). Also, IL-4 and IL-10 were positively correlated (*r* = 0.49, *p* = 0.003). However, only granzyme B correlated negatively with IL-17A (*r* = −0.35, *p* = 0.032). In addition, in children with submicroscopic infections, IL-12p70 correlated negatively with IL-10 (*r* = -0.52, *p* = 0.012), whereas, granzyme B correlated positively with IL-4 (*r* = 0.62, *p* = 0.002) (Supplementary Table S1). However, we observed that using principal component analysis, the cytokine profiles could not differentiate children with either microscopic or submicroscopic asymptomatic malaria from uninfected controls (Figure 3).

**TABLE 2:** Correlation between the pro- and anti-inflammatory cytokines in children with microscopic asymptomatic infections.

	Granzyme B	IFN-γ	TNF-α	IL-6	IL-12 p70	IL-4	IL-10	IL-17A
Granzyme B	1							
IFN-γ	–0.07	1						
TNF-α	–0.11	0.22	1					
IL-6	–0.11	0.15	0.59****	1				
IL-12 p70	0.04	–0.02	−0.19	−0.14	1			
IL-4	–0.07	0.21	0.41*	0.39*	0.30	1		
IL-10	–0.08	–0.25	0.39*	0.50**	0.16	0.47**	1	
IL-17A	−0.35*	0.25	−0.10	0.17	−0.01	0.14	−0.05	1

Significant associations are in bold and denoted by *p < 0.05, **p < 0.01, ****p < 0.0001.

Also, to determine the impact of parasitemia on these cytokines, we initially determined if parasitemia correlated with any of the cytokines measured and only IL-6 correlated significantly with parasitemia (*r* = 0.36, *p* = 0.029; Supplementary Figure S1). Furthermore, using a generalized additive model and a likelihood ratio test to determine the impact of parasitemia on the secretion of these cytokines, parasitemia seems to be a major predictor of IL-10 and IL-6 levels (Table 3).

**TABLE 3:** The association between inflammatory mediators and parasitemia for children with microscopic asymptomatic infection.

Covariates	*P*-value in model	Deviance explained (%)	LR test *p*-value
Granzyme B	0.84	60.3	0.82
IFN-γ	0.96	60.4	0.96
TNF-α	0.84	60.3	0.82
IL-6	**0.019**	51.9	**0.007**
IL-12p70	0.77	60.2	0.73
IL-4	0.22	58.3	0.16
IL-10	**0.004**	47.4	**0.001**
IL-17A	0.98	60.4	0.98

*Significant associations are in bold.*

### Association Between Cytokine Levels and Age

It has been reported that cytokine levels during infection may vary with age (Mshana et al., 1991; Farrington et al., 2017) and since age differed significantly in our study population, we determined whether cytokine responses are impacted by age. It was observed that none of the cytokine levels were significantly associated with age in children with microscopic or submicroscopic malaria infection. However, in a multivariate analysis using a generalized additive model, age was a good predictor of granzyme B (*p* = 0.002) and IL-6 (*p* = 0.04) levels (Supplementary Table S2).

### Pro-inflammatory/Anti-inflammatory Ratio Does Not Differ Between Asymptomatic Children and Uninfected Controls

**FIGURE 4:**
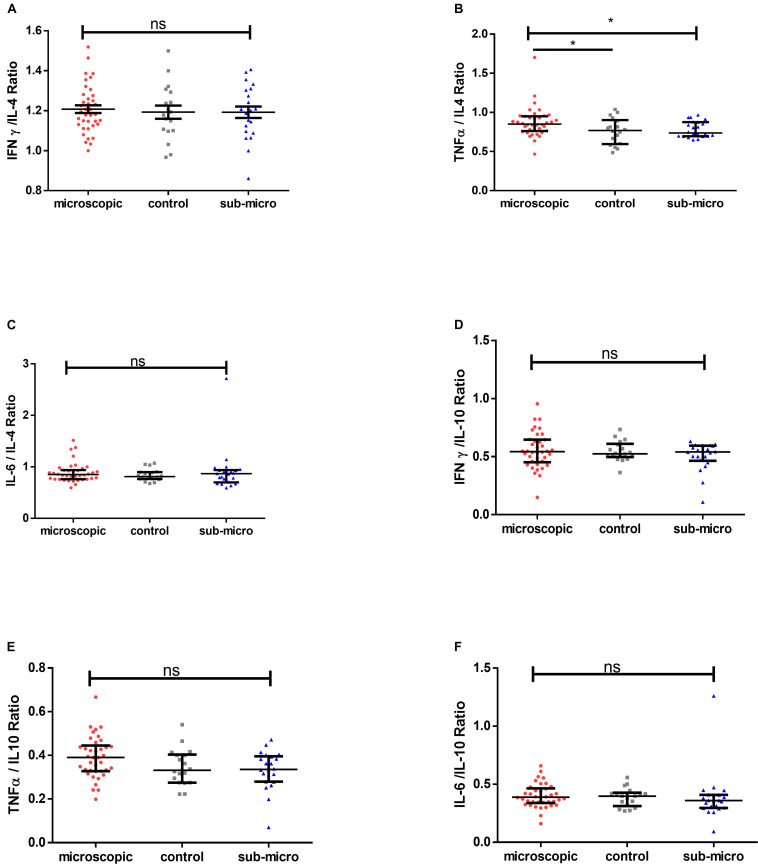
Comparable pro-inflammatory/anti-inflammatory cytokine ratios in microscopic, submicroscopic asymptomatic infected and uninfected controls. The graphs **(A–F)** shows the ratio of IFN-γ/IL-4, TNF-α/IL-4, IL-6/IL-4 and IFN-γ/IL-10, TNF-α/IL-10, IL-6/IL-10 in uninfected controls (*n* = 18), microscopic (*n* = 38), and submicroscopic asymptomatic malaria infection (*n* = 22). Data are displayed as scatter plots showing median and interquartile ranges. Significant values are indicated by *p < 0.05.

To determine whether pro-inflammatory cytokines or regulatory cytokines dominate asymptomatic infections, the ratio of IFN-γ, TNF-α and IL-6 to IL-4 and IL-10 were measured and compared with that in uninfected controls. We first compared the ratio of IFN-γ/IL-4, TNF-α/IL-4, and IL-6/IL-4. There were no significant differences in IFN-γ/IL-4 and IL-6/IL-4 ratios when compared between microscopic or submicroscopic asymptomatic children and controls (*p* > 0.05 in all cases). The only exception was for TNF-α/IL-4 which was significantly increased in children with microscopic asymptomatic malaria compared to uninfected controls using the Mann-Whitney test (*p* = 0.044). Secondly, we also compared the ratio of IFN-γ/IL-10, TNF-α/IL-10, and IL-6/IL-10. It was observed that the ratios of IFN-γ/IL-10, TNF-α/IL-10, and IL-6/IL-10 did not differ between children with microscopic asymptomatic malaria, submicroscopic asymptomatic malaria and uninfected controls (Figure 4). Thus, although most cytokine levels were increased in response to infection, the balance between pro- and anti-inflammatory cytokines was largely maintained in the infected group when compared to the uninfected controls.

## Discussion

Asymptomatic malaria remains a major challenge in malaria control elimination programs due to its significant impact on disease transmission (Alves et al., 2005; Schneider et al., 2007). Even though it has been noted that asymptomatic malaria may confer partial immunity against malaria (Tran et al., 2013), it can also be a precursor to the development of clinical disease (Njama-Meya et al., 2004). Cytokines produced by different cell types are the normal expected immune response against invading pathogens but can also drive immunopathology. Various studies have shown that a disproportionate increase in the levels of pro-inflammatory cytokines can lead to an immune-associated pathology and cause the progression of asymptomatic infections to febrile and severe forms (Othoro et al., 1999; Jagannathan et al., 2014; Mandala et al., 2017). In this study, we determined the impact of asymptomatic malaria infections on the secretion of pro- and anti-inflammatory cytokines and how this may affect disease outcome. We observed increased levels of cytokines such as TNF-α and IL-6 in children with microscopic asymptomatic malaria, whereas, IFN-γ, IL-17A, and IL-4 levels were increased in infected children with microscopic or submicroscopic asymptomatic malaria compared with uninfected controls. Also, levels of granzyme B were decreased in children with either microscopic or submicroscopic infections compared to uninfected controls whilst levels of IL-10 and IL-12p70 were comparable between infected and uninfected children.

TNF-α induces reactive oxygen species, cell death and the secretion of other cytokines such as IL-1 and IL-6. In addition, it regulates the production of IL-12 by macrophages, primes neutrophils and serves as a cofactor for IL-12 induced IFN-γ production (Malaguarnera and Musumeci, 2002). Studies have shown that increasing levels of TNF-α and IL-6 in malaria are associated with the cytoadherence of infected erythrocytes leading to the development of febrile disease (Lyke et al., 2004; Robinson et al., 2009; Cruz et al., 2016). Also, increasing IL-6 levels mediates the production of acute-phase reactant proteins such as C-reactive proteins (CRP) and secretory phospholipase A2 (sPLA2) (Juffrie et al., 2001) whereas moderate levels can reduce parasitemia (Lyke et al., 2004). However, it remains to be understood whether TNF-α association with immune pathology is due to its ability to induce IL-1, IL-6, and IFN-γ secretion. In addition, others have reported moderate levels of TNF-α to be associated with parasite control by stimulating monocyte to phagocytose infected erythrocytes as well as activate calcium signaling in human malaria parasites (Aggarwal, 2003; Cruz et al., 2016; Oyegue-Liabagui et al., 2017). In our study, we observed that children with microscopic infections had increased levels of TNF-α and IL-6 compared to uninfected controls and children with submicroscopic infections. Probably denoting that with increasing parasitemia, TNF-α as well as IL-6 may work synergistically to decrease parasitemia levels.

Protective mechanisms induced by IFN-γ have been reported during liver-stage and blood-stage infections (D’Ombrain et al., 2008; Robinson et al., 2009) with predominant sources of this cytokine being T cells and NK cells (Dodoo et al., 2002; D’Ombrain et al., 2008; Jagannathan et al., 2017). In addition, Increasing levels of IL-17A have been associated with inflammation in malaria and mediating protection in various infectious diseases through the recruitment of immune cells such as neutrophils and mediate the production of several pro-inflammatory cytokines (Kelly et al., 2005; Oyegue-Liabagui et al., 2017). Here, we show that asymptomatic infection with *Plasmodium* is associated with higher levels of IFN-γ and IL-17A. However, neither of these cytokines correlated with age or parasitemia. Nonetheless, the increased levels observed in the asymptomatic malaria group may indicate that IFN-γ and IL-17A are associated with on-going inflammation which has a significant impact on disease pathogenesis as well as the development of anti-disease immunity.

It has previously been shown that asymptomatic infections are characterized by limited T cell activation and regulation (Frimpong et al., 2018). Likewise, levels of regulatory T cells (Tregs) (Boyle et al., 2015) and activated Tregs did not differ significantly between children with asymptomatic malaria and uninfected controls (Frimpong et al., 2018). Interestingly, other studies have observed that individuals with asymptomatic *P. vivax* infections had lower levels of the Treg cytokine IL-10 compared to controls (Jangpatarapongsa et al., 2008; Bueno et al., 2012) whereas, a study by Othoro et al. (1999) found no significant difference in levels between asymptomatic cases and uninfected controls. Also, IL-10 levels have been noted to decrease with increasing age (Boyle et al., 2017). Therefore, the lack of significant difference observed in the levels of IL-10 in the current study may be as a result of the age differences. Probably, it could indicate that IL-10 may not be the sole anti-inflammatory cytokine in mediating inflammation in asymptomatic malaria and its production may be transient during asymptomatic malaria. It may also suggest that IL-10 production is directly affected by levels of parasitemia since IL-10 was the only anti-inflammatory cytokine that was a good predictor of parasitemia and it may be secreted in a dose dependent manner. This could also imply that increasing IL-10 levels may be associated with the development of febrile disease (Farrington et al., 2017; Oyegue-Liabagui et al., 2017). Even though both IL-10 and IL-12p70 levels did not differ among the study population, they were negatively correlated in children with submicroscopic infections indicating the inhibitory effect of IL-10 on IL-12p70.

On the other hand, IL-4 was found to increase significantly in children with microscopic and submicroscopic malaria compared to controls. IL-4 like IL-10 has an important role in immunoregulation by downregulating the secretion and activity of pro-inflammatory cytokines like TNF-α, IL-6, and IL-17A (Riley et al., 2006). The positive relationship between IL-4 and IL-10 and these pro-inflammatory cytokines (TNF-α and IL-6) in children with microscopic infections, supports the counter-regulatory activity of these anti-inflammatory cytokines on TNF-α and IL-6. Also, IL-4 has been reported to suppress the upregulation of granzyme B in T cells, thereby reducing induced cell death (Riou et al., 2006). Additionally, decreasing levels of granzyme B has been associated with a Th2 response (Devadas et al., 2006), whereas, increasing levels in malaria have been observed in children with severe and uncomplicated infections (Kaminski et al., 2019). Here the lower levels of granzyme B observed in children with malaria infection and the upregulation of IL-4 support the immunoregulatory activity of IL-4 on granzyme B. Furthermore, the positive correlation observed between both IL-4 and granzyme B in children with submicroscopic infections may indicate that probably at the sub-patent level of infection, the effect of IL-4 on regulating granzyme B expression is reduced. Likewise, the negative correlation observed between Granzyme B and IL-17A may have resulted from the low levels of granzyme B observed in malaria-infected children.

The outcome of various diseases in humans may largely be based on a pro- or anti-inflammatory response balance, since these produce cytokines with diverse functions and outcome. Asymptomatic malaria has been proposed to be associated with anti-inflammatory responses (Ochola-Oyier et al., 2019). We tested the hypothesis whether there is a polarization toward immunoregulatory cytokines during asymptomatic infection. The data, however, demonstrated that both pro-inflammatory and anti-inflammatory cytokines are upregulated in asymptomatic infections, however, the ratios of pro-inflammatory/anti-inflammatory responses were comparable between the study groups. This may suggest that a balance between pro- and anti-inflammatory cytokines may be responsible for the absence of clinical disease symptoms despite the presence of parasites.

The study had some limitations since it was cross-sectional and participants were sampled at a single time point. Also, participants were not followed to determine if any of them progressed to febrile disease and to assess trends in the cytokine profile during asymptomatic infection and disease onset in the same individuals. Also, the presence of other infectious diseases such as helminth co-infections which were unaccounted for may account for some of the differences in the cytokine profile since they are common in children of school-going age (Brooker et al., 2007; Salazar-Castañón et al., 2018). Nevertheless, we have been able to show that asymptomatic malaria infections are characterized by a concomitant upregulation of both pro- and anti-inflammatory cytokines, specifically TNF-α, IFN-γ, IL-6, IL-17A, and IL-4 as assessed in this study. The observed maintenance of a balance between pro- and anti-inflammatory mediators may in part explain the asymptomatic status of infected children. Thus, disease symptoms will only develop when there is a perturbation of this balance, especially toward an increased pro-inflammatory response which has been severally shown to have immunopathological consequences.

## Conclusion

The data show that despite the increase in both pro- and anti-inflammatory mediators in children with asymptomatic microscopic and submicroscopic infections, there is a homeostatic maintenance of the balance between pro- and anti-inflammatory cytokines. This outcome is very consistent with the numerously reported observations that an imbalance in the levels of pro- and anti-inflammatory cytokines are an essential trigger of febrile disease in infected persons.

## Data Availability Statement

The raw data supporting the conclusions of this article will be made available by the authors, without undue reservation.

## Ethics Statement

The studies involving human participants were reviewed and approved by the Institutional Review Board of the Noguchi Memorial Institute for Medical Research at the University of Ghana. Written informed consent to participate in this study was provided by the participants’ legal guardian/next of kin.

## Author Contributions

KK and AF conceived the idea and designed the experiments and supervised the work. JA, AA, DA, and LB-E performed the experiments in the study and were assisted by EO, EK-B, and KA-M. AF, BA, GM, LA, and KK wrote the manuscript. All authors read and approved the final manuscript.

## Conflict of Interest

The authors declare that the research was conducted in the absence of any commercial or financial relationships that could be construed as a potential conflict of interest.
