# Corrigendum: Asymptomatic Malaria Infection Is Maintained by a Balanced Pro- and Anti-inflammatory Response

**DOI:** 10.3389/fmicb.2021.686435

**Published:** 2021-05-19

**Authors:** Augustina Frimpong, Jones Amponsah, Abigail Sena Adjokatseh, Dorothy Agyemang, Lutterodt Bentum-Ennin, Ebenezer Addo Ofori, Eric Kyei-Baafour, Kwadwo Akyea-Mensah, Bright Adu, Gloria Ivy Mensah, Linda Eva Amoah, Kwadwo Asamoah Kusi

**Affiliations:** ^1^West African Centre for Cell Biology of Infectious Pathogens (WACCBIP), University of Ghana, Accra, Ghana; ^2^Department of Immunology, Noguchi Memorial Institute for Medical Research, College of Health Sciences, University of Ghana, Accra, Ghana; ^3^African Institute for Mathematical Sciences, Accra, Ghana; ^4^Department of Biochemistry, Cell and Molecular Biology, College of Basic and Applied Sciences, University of Ghana, Accra, Ghana; ^5^Department of Bacteriology, Noguchi Memorial Institute for Medical Research, College of Health Sciences, University of Ghana, Accra, Ghana

**Keywords:** microscopic, *Plasmodium*, anti-inflammatory cytokines, pro-inflammatory cytokines, asymptomatic malaria, submicroscopic

In the original article, there was a mistake in Figure 1C as published. The graph provided was a duplicate of Figure 1E. The corrected Figure 1 and caption appear below.

**Figure 1:**
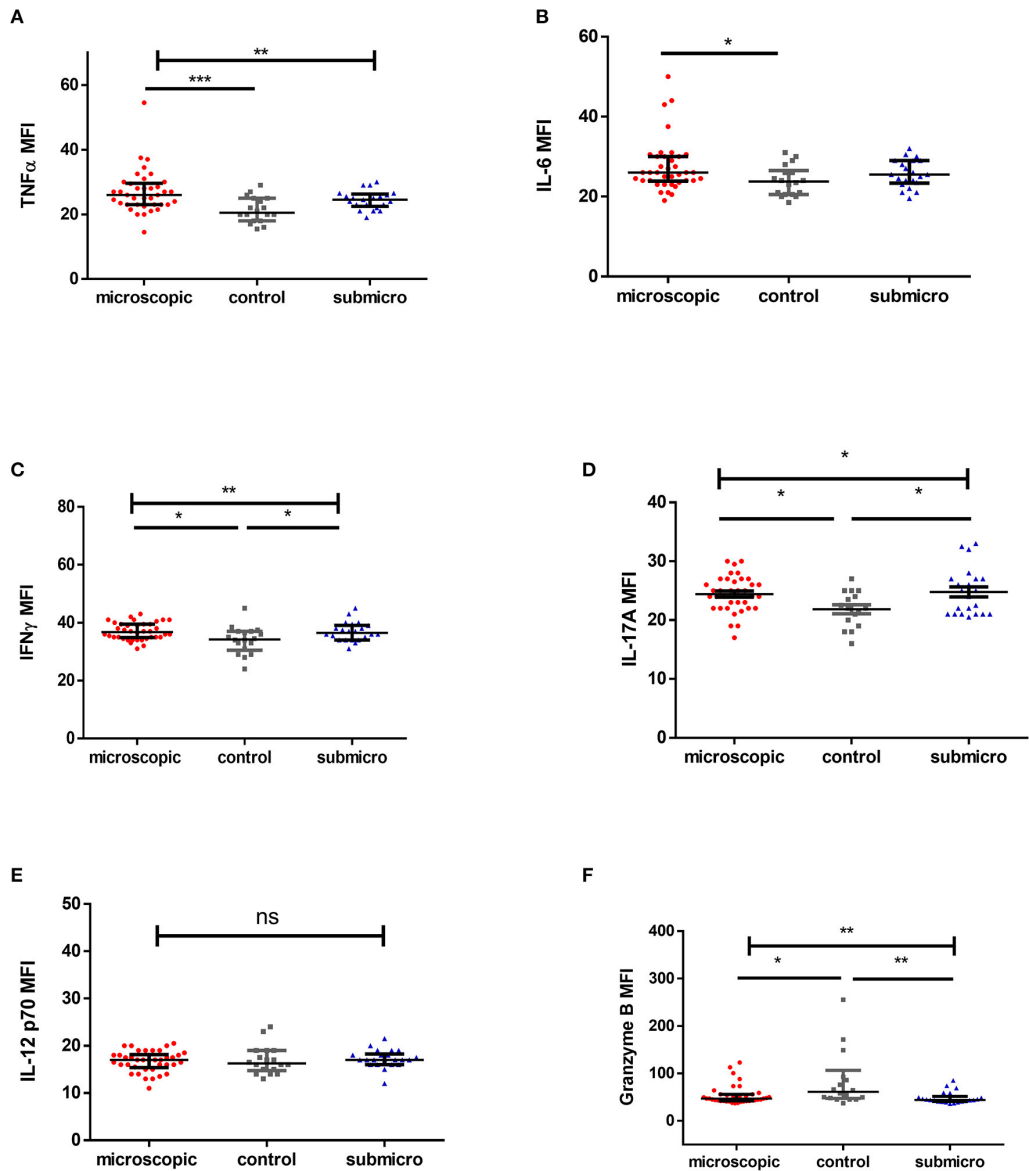
Profile of pro-inflammatory mediators during microscopic and submicroscopic malaria. Scatter plot graphs are plotted showing the median fluorescence intensities (MFI) of **(A–F)** TNF-α, IL-6, IFN-γ, IL-17A, IL-12p70, and Granzyme B in plasma samples collected from uninfected controls (*n* = 18), patients with microscopic asymptomatic malaria (*n* = 38) and submicroscopic malaria (*n* = 22). Plots show median and interquartile ranges. Significant differences are denoted by *p < 0.05, **p < 0.01, ***p < 0.001, ns = not significant.

The authors apologize for this error and state that this does not change the scientific conclusions of the article in any way. The original article has been updated.

