# Supplementary figures and images for: Asymptomatic Malaria Infection Is Maintained by a Balanced Pro- and Anti-inflammatory Response
Source: Front Microbiol. 2020 Nov 17;11:559255. doi: 10.3389/fmicb.2020.559255 (PMC7705202; doi:10.3389/fmicb.2020.559255)

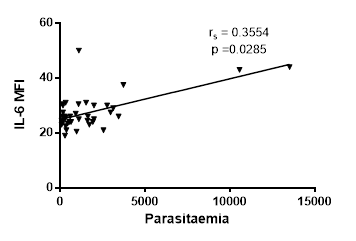

Supplement: Supplementary file 1 [file Image_1.JPEG]
