# Supplementary material for: Asymptomatic Malaria Infection Is Maintained by a Balanced Pro- and Anti-inflammatory Response
Source: Front Microbiol. 2020 Nov 17;11:559255. doi: 10.3389/fmicb.2020.559255 (PMC7705202; doi:10.3389/fmicb.2020.559255)
Supplement: Supplementary file 2 [file Table_1.docx]

# Supplementary Table 1

**Table S1:** A Spearman rank correlation coefficient denoting the relationship between the pro- and anti-inflammatory mediators for children with sub-microscopic asymptomatic malaria infection

|  | Granzyme B | IFN-γ | TNF-α | IL-6 | IL-12 p70 | IL-4 | IL-10 | IL-17A |
| --- | --- | --- | --- | --- | --- | --- | --- | --- |
| Granzyme B | 1 |  |  |  |  |  |  |  |
| IFN-γ | -0.17 | 1 |  |  |  |  |  |  |
| TNF-α | 0.06 | **-0.60**** | 1 |  |  |  |  |  |
| IL-6 | 0.28 | 0.13 | -0.20 | 1 |  |  |  |  |
| IL-12 p70 | -0.04 | 0.11 | 0.09 | -0.15 | 1 |  |  |  |
| IL-4 | **0.62**** | 0.02 | 0.15 | -0.14 | 0.01 | 1 |  |  |
| IL-10 | 0.12 | -0.08 | -0.34 | 0.14 | **-0.52*** | -0.07 | 1 |  |
| IL-17A | 0.03 | -0.31 | 0.08 | 0.02 | 0.38 | 0.15 | -0.13 | 1 |

Significant values are in bold and indicated by *p<0.05, **p<0.01
