# Supplementary material for: Asymptomatic Malaria Infection Is Maintained by a Balanced Pro- and Anti-inflammatory Response
Source: Front Microbiol. 2020 Nov 17;11:559255. doi: 10.3389/fmicb.2020.559255 (PMC7705202; doi:10.3389/fmicb.2020.559255)
Supplement: Supplementary file 3 [file Table_2.docx]

# Supplementary Table 2

**Table S2:** The association between inflammatory mediators and age for children with submicroscopic asymptomatic infection.

| **Covariates** | **P-value in model** | **Deviance explained (%)** | **LR test p-value** |
| --- | --- | --- | --- |
| Granzyme B | **0.008** | 22.2 | **0.002** |
| IFN-γ | 0.23 | 36 | 0.17 |
| TNF-α | 0.92 | 39 | 0.91 |
| IL-6 | 0.08 | 31.9 | **0.040** |
| IL-12p70 | 0.71 | 38.8 | 0.67 |
| IL-4 | 0.69 | 38.7 | 0.64 |
| IL-10 | 0.88 | 39 | 0.86 |
| IL-17A | 0.52 | 38.2 | 0.46 |

Significant values are in bold.
